# The impact of ischemic stroke on connectivity gradients

**DOI:** 10.1016/j.nicl.2019.101947

**Published:** 2019-07-19

**Authors:** Şeyma Bayrak, Ahmed A. Khalil, Kersten Villringer, Jochen B. Fiebach, Arno Villringer, Daniel S. Margulies, Smadar Ovadia-Caro

**Affiliations:** a Department of Neurology, Max Planck Institute for Human Cognitive and Brain Sciences, Leipzig, Germany; b Department of Cognitive Neurology, University Hospital Leipzig and Faculty of Medicine, University of Leipzig, Leipzig, Germany; c Center for Stroke Research Berlin, Charité - Universitätsmedizin Berlin, Berlin, Germany; d Berlin School of Mind and Brain, Humboldt-Universität zu Berlin, Berlin, Germany; e Centre National de la Recherche Scientifique (CNRS) UMR 7225, Frontlab, Institut du Cerveau et de la Moelle épinière, Paris, France; f Department of Neurology, Campus Benjamin Franklin, Charité - Universitätsmedizin Berlin, Berlin, Germany

**Keywords:** Connectivity gradients, Intrinsic functional connectivity, Diaschisis, Resting-state fMRI, Connectome, Diffusion embedding

## Abstract

The functional organization of the brain can be represented as a low-dimensional space that reflects its macroscale hierarchy. The dimensions of this space, described as connectivity gradients, capture the similarity of areas' connections along a continuous space. Studying how pathological perturbations with known effects on functional connectivity affect these connectivity gradients provides support for their biological relevance. Previous work has shown that localized lesions cause widespread functional connectivity alterations in structurally intact areas, affecting a network of interconnected regions. By using acute stroke as a model of the effects of focal lesions on the connectome, we apply the connectivity gradient framework to depict how functional reorganization occurs throughout the brain, unrestricted by traditional definitions of functional network boundaries. We define a three-dimensional connectivity space template based on functional connectivity data from healthy controls. By projecting lesion locations into this space, we demonstrate that ischemic strokes result in dimension-specific alterations in functional connectivity over the first week after symptom onset. Specifically, changes in functional connectivity were captured along connectivity Gradients 1 and 3. The degree of functional connectivity change was associated with the distance from the lesion along these connectivity gradients (a measure of functional similarity) regardless of the anatomical distance from the lesion. Together, these results provide support for the biological validity of connectivity gradients and suggest a novel framework to characterize connectivity alterations after stroke.

## Highlights

• Impact of stroke follows continuous architecture of connectivity gradients.
• The widespread effects of stroke can be detected at the connectome level.
• Distance from lesion within connectivity space captures changes during recovery.
• Connectivity gradients offer template to capture reorganization at connectome level.

## 1 Introduction

The assessment of functional connectivity based on the temporal correlation of ongoing blood-oxygen-level-dependent (BOLD) fluctuations (resting-state functional magnetic resonance imaging; rs-fMRI) has transformed our understanding of the brain's reorganization and recovery after injury (Carter et al., 2012; Fornito and Bullmore, 2010, Fornito and Bullmore, 2015; Fox, 2010; Gillebert and Mantini, 2013; Zhang and Raichle, 2010). Functional networks are usually defined as discrete entities comprised of brain regions sharing similar features and being strongly connected with each other. Such an approach assumes that there are sharp boundaries between areas representing different functional domains and that connectivity within a given network is homogenous. These assumptions are useful for understanding the brain's functional organization. However, recent advances take us further, enabling us to capture additional key characteristics of how these functional domains are organized, namely as topographical or hierarchical *processing gradients* (Atasoy et al., 2016; Cerliani et al., 2012; Haak et al., 2018).

Recently, non-linear decomposition of rs-fMRI data was introduced as a method to capture these features by representing whole-brain connectivity in a continuous, low-dimensional space. This data-driven analysis results in *connectivity gradients* that provide a description of the connectome where each voxel is located along a connectivity gradient according to its connectivity pattern (Langs et al., 2014, Langs et al., 2016; Margulies et al., 2016). Voxels that are similar in terms of connectivity patterns are situated close to one another along a given connectivity gradient (Huntenburg et al., 2018). Different functional modules are thus distributed along these gradients (Krienen and Sherwood, 2017) in a manner that reflects the hierarchical organization of brain function at the macroscale level (Margulies et al., 2016). This provides a novel framework for describing multiple large-scale networks in a continuous and biologically plausible manner (Mesulam, 2012). In order to support the use of gradients to study brain organization, investigating the effects of perturbations on gradients is essential.

Stroke is a powerful model for studying the influence of localized changes on the brain's functional organization (de Haan and Karnath, 2018; Karnath et al., 2018). Although the initiating event is a localized injury to the central nervous system, areas outside (but functionally connected to) this lesion undergo functional alterations that are implicated in stroke symptomology and the recovery from neurological deficits (Corbetta, 2010; Ovadia-Caro et al., 2014; Ward, 2005). This phenomenon is known as *diaschisis* (Andrews, 1991; Carrera and Tononi, 2014) and provides a theoretical and empirical motivation to study brain connectivity following stroke. In addition, stroke can induce a localized injury virtually anywhere in the brain, allowing the effects of damage to different brain regions on the brain's functional organization to be investigated in the search for a mutual basis for reorganization mechanisms.

By studying discrete networks, functional connectivity has been used to detect functional reorganization after stroke within the affected domain. Reduction in functional connectivity is associated with the severity of the clinical deficit (Baldassarre et al., 2014; Carter et al., 2010; He et al., 2007; Ovadia-Caro et al., 2013; Siegel et al., 2016a, Siegel et al., 2016b; Wang et al., 2010; Warren et al., 2009) and normalization of functional connectivity patterns was found following both spontaneous post-stroke recovery (He et al., 2007; Park et al., 2011; Ramsey et al., 2016; van Meer et al., 2010) and interventions using non-invasive brain stimulation (Volz et al., 2016).

**Fig. 1:**
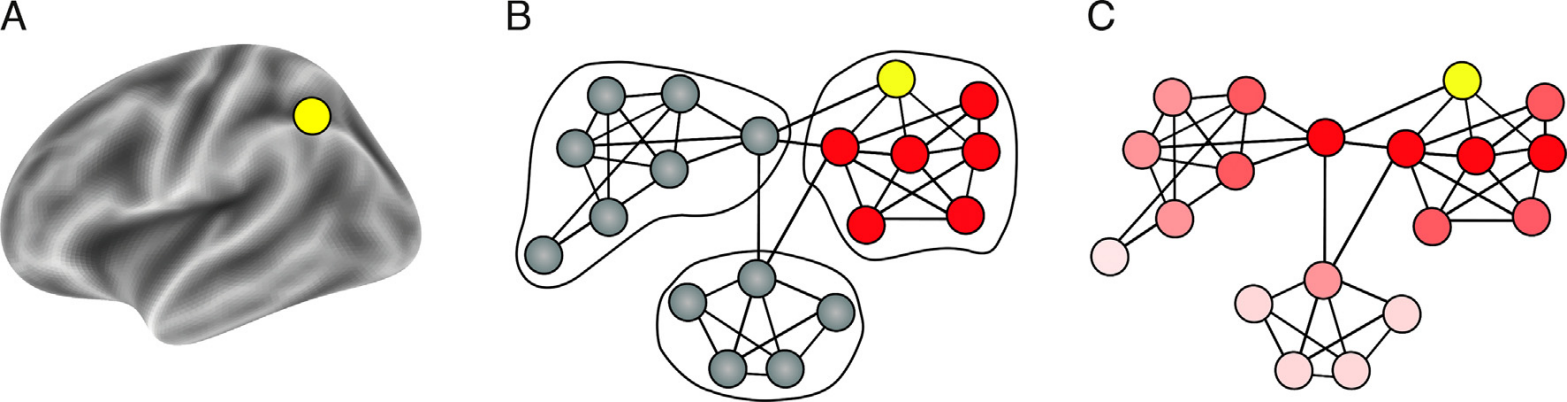
Two complementary views on brain organization and the corresponding representation of distal effects of focal lesions. (A) A focal lesion (yellow node) on the brain anatomical surface. (B) A schematic description of discrete network parcellation superimposed on a functional connectivity graph-space with nodes and edges. Using this approach to study the effects of focal lesions (yellow node) restricts us to singular networks and assigns connector hubs to one network only. Additionally, distal effects of the lesion are assumed to be equally disruptive for all nodes in the affected network (red nodes). (C) Representing functional connectivity in a continuous manner without sharply defined borders using connectivity gradients. The lesioned node affects all other nodes in the system as a function of the distance from the lesion in connectivity space (dark red to light red). Using this approach does not assume sharp boundaries between functional networks and provides a more realistic model of distant effects of localized lesions.

While previous studies demonstrate the role of the affected network in stroke pathology, several lines of evidence suggest that the impact of a lesion is not limited by discrete network definitions. It is well known that the effects of stroke within a given network are not necessarily uniformly or homogenously distributed (Grefkes and Fink, 2014; Rehme and Grefkes, 2013). In addition, computational models of brain connectivity demonstrate that the disruption of a single node extends beyond the affected network and impacts, to varying degrees, the whole graph (Aerts et al., 2016). Lastly, the extent and nature of the resulting disturbance to functional organization depends on the topological role of the lesioned area. Damage to connector hubs, which connect different sub-networks, results in more severe disturbance to network organization (Gratton et al., 2012) and more severe cognitive deficits (Warren et al., 2014) compared with damage to other regions. Connector hub damage results in reduction in modularity (Gratton et al., 2012), defined as the degree to which the connectome is organized into highly clustered local modules. Importantly, the recovery of modularity has been associated with clinical improvement after stroke (Siegel et al., 2017) thereby emphasizing the importance of hubs to the reorganization process. Methods that discretize functional networks necessarily allocate connector hubs to a single sub-network, thereby misrepresenting their integrative role. These limitations, which are a necessary by-product of discrete approaches to functional connectivity, can be overcome using continuous approaches (Fig. 1).

Here, we study the impact of stroke on a continuous template representing functional connectivity at the voxel-level. Data from healthy subjects were used to create a template of three connectivity gradients representing all possible connections in a continuous manner (connectivity space). The effects of stroke on this continuous template were quantified using longitudinal rs-fMRI data from patients starting within 24 h of symptom onset.

Based on previous findings in discrete networks (Baldassarre et al., 2014; Carter et al., 2010; He et al., 2007; Nomura et al., 2010; Ovadia-Caro et al., 2013; Siegel et al., 2016a, Siegel et al., 2016b; Wang et al., 2010; Warren et al., 2009) and computational models (Alstott et al., 2009; Honey and Sporns, 2008; van Dellen et al., 2013; Young et al., 2000), we hypothesized that a lesion in continuous connectivity space would induce a gradual impact on the whole connectome and that this would be most pronounced in areas that share a similar functional connectivity pattern with the lesion.

## 2 Materials and methods

### 2.1 Participants

Fifty-four stroke patients (20 females, age: 63.78 ± 12.03 years, mean ±SD) and 31 healthy controls (13 females, age: 64.90 ± 8.49 years) were initially recruited for the study. Inclusion criteria for patients were: age > 18 years, first ever ischemic stroke – small cortical (≤1.5 cm) or subcortical, which was evident in imaging, Wahlund score ≤ 10 (Wahlund et al., 2001) to limit the extent of white matter lesions. Exclusion criteria included: history of previous stroke (*n* = 3), fewer than 3 resting-state scans post-stroke (*n* = 10), lesions located solely within white matter (n = 3 patients), corrupted MRI raw data or distorted images (n = 1 control, *n* = 4 patients), high degree of head motion (n = 1 control, *n* = 6 patients), and poor registration quality (n = 1 control). For further details on quality assessment, see Supplementary Material M1.

Following the exclusion procedure, 28 stroke patients (11 females, age: 65.04 ± 13.27 years, mean ±SD), and 28 healthy controls (13 females, age: 65.21 ± 8.84 years) were included in the analysis. No significant differences between the groups were found for age and sex (age: Welch's *t*-test, *P* = .95; sex: Kruskal-Wallis H-test, *P* = .59). For further details on patients' information, see Supplementary Table 1. The study was approved by the ethics committee of the Charité - Universitätsmedizin Berlin, Germany (EA 1/200/13). Written informed consent was obtained from all participants.

### 2.2 Neuroimaging data

The MRI protocol included T1-weighted structural scans and T2*-weighted resting-state fMRI scans (continuous fMRI scan with no overt task) for all participants. In addition, diffusion weighted images (DWI; TR = 8.2 s, TE = 0.1 s, 50 volumes, voxel size: 2 × 2 × 2.5 mm, flip angle 90°) and fluid attenuated inversion recovery images (FLAIR; TR = 8.0 s, TE = 0.1 s, 54 volumes, voxel size: 0.5 × 0.5 × 5 mm) were acquired from the stroke patients as part of a standard MRI protocol (Hotter et al., 2009). All MRI data were acquired on a Siemens Tim Trio 3 T scanner. Healthy control participants were scanned at a single time point, whereas stroke patients were scanned at three consecutive time points relative to stroke symptom onset: day 0 (within 24 h), day 1 (24–48 h), and day 5 (range: day 4–6, mean 4.93 ± 0.38 SD). Structural scans were acquired using a three-dimensional magnetization prepared rapid gradient-echo (MPRAGE) sequence (TR = 1.9 s, TE = 2.52 s, TI = 0.9 s, 192 slices, voxel size: 1 × 1 × 1 mm, flip angle 9°). Resting-state functional scans for each participant and session were acquired using blood-oxygenation-level-dependent (BOLD) contrast with an EPI sequence (TR = 2.3 s, TE = 0.03 s, 34 slices, 150 volumes, voxel size: 3 × 3 × 3 mm, flip angle 90°, total duration = 5.75 min).

### 2.3 Data preprocessing

All image processing and statistical analysis scripts used in this study are available at https://github.com/sheyma/stroke_preprop.

T1-weighted structural images were preprocessed using FreeSurfer's recon-all pipeline (v6.0.0, (Dale et al., 1999)). The pipeline generated segmentations for grey matter, white matter and cerebrospinal fluid. Individual grey matter masks were registered to standard MNI space (3 mm^3^).

Preprocessing of functional images included: *i)* removal of the first 5 EPI volumes to avoid signal saturation, *ii)* slice timing and motion correction (Nipype v0.14.0, (Gorgolewski et al., 2011; Roche, 2011)), *iii)* CompCor denoising approach for time series at the voxel-level (Nilearn v0.4.0, (Behzadi et al., 2007)), *iv)* temporal normalization, *v)* band-pass filtering in the range of 0.01–0.1 Hz, and *vi)* spatial smoothing (applied after registration) with a 6 mm full-width-half maximum Gaussian kernel using FSL (v5.0.9, (Woolrich et al., 2009)). Confounds removed from the time series at the denoising step were defined as *i)* six head motion parameters, including 1st and 2nd order derivatives, *ii)* motion and intensity outliers (Nipype's rapidart algorithm; thresholds: > 1 mm framewise head displacement, and signal intensity >3 SD of global brain signal accordingly) and *iii)* signal from white matter and cerebrospinal fluid.

The transformation of functional images to MNI152 (3 mm^3^) space included a linear transformation from EPI to the high-resolution T1-weighted image using FreeSurfer's boundary-based register tool with 6 degrees of freedom (Greve and Fischl, 2009) and a nonlinear transformation using ANTs (v2.1.0, (Avants et al., 2011)). The transformation matrices obtained from both steps were concatenated and applied to the functional image using a single interpolation step.

### 2.4 Lesion delineation

Lesions were manually delineated by identifying areas of localized hyperintensity on day 0 DWI images using the ITK-SNAP software (v3.4.0, (Yushkevich et al., 2006)). Delineations were done by the first author (Ş.B.) and checked by a researcher with 6 years' experience in stroke imaging (A.K.). All lesion masks were normalized to MNI152 (3 mm^3^) space (ANTs, nearest-neighbor interpolation). Individual lesion masks were smoothed in the atlas space using FSL's dilation tool with 3 × 3 × 3 kernel, extending the mask by one voxel-size (v5.0.9, (Jenkinson et al., 2012)).

### 2.5 Computing connectivity gradients by applying nonlinear decomposition to functional connectivity data from healthy controls

To create a mutual grey matter template to be used for decomposition analysis, individual grey matter masks and resting-state functional masks were averaged for all healthy controls to create a group mask (for a detailed description, see Supplementary material M2). In brief, averaged group maps were multiplied to create a mutual mask such that only grey matter voxels would be included. The resulting template (33,327 voxels) was used to generate functional connectivity matrices from individual healthy controls.

**Fig. 2:**
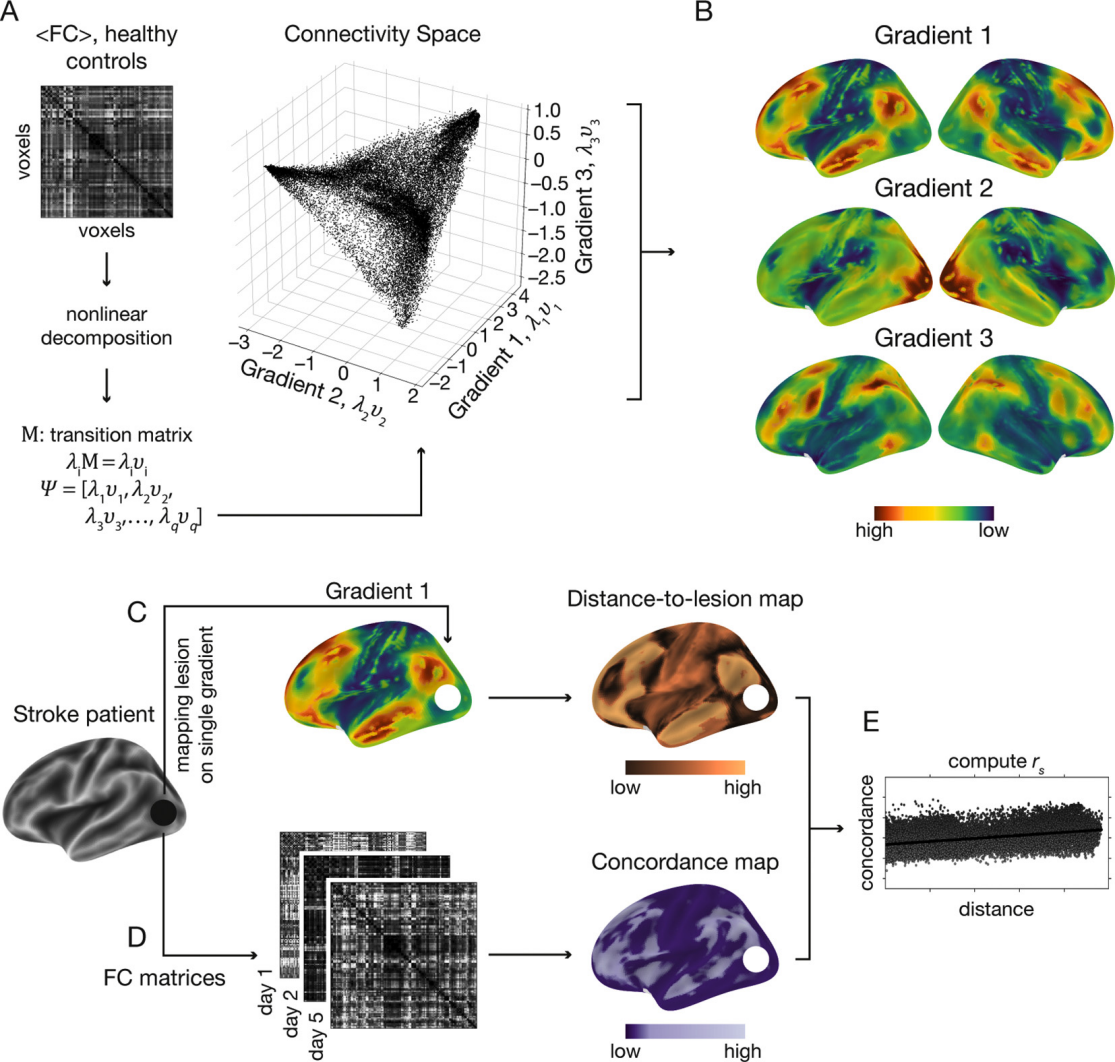
A schematic description of the analysis steps. (A) Averaged functional connectivity matrix (<FC>) based on the resting-state fMRI data of 28 healthy subjects. As an alternative to parcellation approaches, the functional connectivity matrix was decomposed into a low dimensional representation using the diffusion embedding algorithm. The scatter plot shows the first 3 eigenvectors (i.e., gradients) used for further analysis: Gradient 1 (x-axis), 2 (y-axis) and 3 (z-axis). Values on each axis depict the embedding values. Voxels are arranged along each dimension based on the similarity of their connectivity pattern, with voxels sharing similar connectivity patterns sharing similar embedding values. (B) Embedding values along single gradients are overlaid on the brain surface to visualize the dissociation they capture. Along Gradient 1, transmodal areas (default-mode network, red) share similar embedding values. At the other extreme of Gradient 1, unimodal sensory areas (blue) share similar embedding values. Gradient 1 therefore represents a dissociation between transmodal and unimodal areas on its two extremes. Gradient 2 depicts the dissociation between the visual network (red), and sensorimotor networks (green-blue). Gradient 3 depicts the dissociation between attention/memory networks (red) and default-mode network as well as sensorimotor network (green-blue). (C) Individual lesions were delineated and located along gradients. An example of a lesion located in the left occipital lobe is shown here (black circle). The distance from each voxel and the lesioned site was computed to create a voxelwise map reflecting the similarity of each voxel's connectivity pattern with that of the lesion for each gradient (“distance-to-lesion” map). This was done by subtracting the embedding values between each voxel and the mean embedding value of the lesioned voxels. Voxels with lower values on the distance map (dark copper) share similar functional connectivity patterns with the lesioned site as characterized in healthy controls. (D) Voxelwise functional connectivity matrices (FC matrices) were computed for the three-consecutive resting-state fMRI scans following stroke onset. Concordance was used to quantify changes in functional connectivity patterns over time. Lower concordance values (dark purple) reflect a larger change in functional connectivity patterns over time. (E) For each gradient and each individual patient, Spearman's rank correlation coefficient (*r*_*s*_) was used to test the relationship between the voxel's connectivity similarity with the lesion and degree of change in functional connectivity over time. The lesioned voxels were excluded from this analysis to capture indirect, rather than local, effects of the lesion. A positive correlation reflects a larger change in functional connectivity over time for voxels that were closer to the lesion site along the corresponding connectivity gradient.

Functional connectivity matrices (33,327 × 33,327 voxels) were computed using Pearson's correlation coefficient and were normalized using Fisher's z-transformation. An average functional connectivity matrix was computed across healthy controls and the averaged z-scores were transformed back to r-scores (Fig. 2A). Each row of the group-level functional connectivity matrix was thresholded at 90% of its r-scores. This yielded an asymmetric, sparse matrix. The pairwise cosine similarities of all rows were computed. By doing this, we obtained a non-negative and symmetric similarity matrix, *L* (values in [0, 1] range).

We implemented the diffusion embedding approach on the similarity matrix to obtain a low-dimensional representation of the whole-brain functional connectivity matrix (Coifman and Lafon, 2006; Langs et al., 2016), as done in Margulies et al., 2016. This approach resulted in gradients of functional connectivity (Fig. 2B). Voxels along each gradient are assigned unitless embedding values. Along each gradient, voxels that share similar connectivity pattern have similar embedding values.

### 2.6 Mapping individual stroke lesions onto connectivity gradients from healthy controls

Individual lesion masks were projected onto the individual gradients obtained in healthy controls. Lesioned voxels were marked according to their location along a specific gradient. The lesion site along each gradient was defined as the minimum embedding value of all lesioned voxels.

To quantify the similarity between non-lesioned voxels and the lesion site in terms of connectivity patterns, distance-to-lesion maps were computed for each non-lesioned voxel (Fig. 2C). Values in these maps reflect the mutual difference between embedding values of non-lesioned and lesioned voxels. Low distance values reflect voxels that share similar functional connectivity pattern with the lesion site.

### 2.7 Quantifying longitudinal changes in functional connectivity matrices for stroke patients

For each patient, a functional mask was obtained from each of the three consecutive functional scans. These masks were multiplied with the grey matter template of the healthy cohort. The dilated lesion segmentations were then excluded from the patient-specific grey matter template. This approach ensured that functional images of patients included only identical grey matter voxels as healthy controls, except for the lesion site. The patient-specific grey matter templates varied slightly in number of voxels included (ranging from 32,659 to 33,212 voxels).

To control for the slight variation in the number of voxels in patient-specific grey matter templates, a control analysis was applied such that the grey matter template used for the analysis contained 30,314 voxels in all patients prior to lesion removal. Using this more restricted mask had no influence on our main results (see Supplementary Material M3 and Supplementary Fig. S1).

Functional connectivity matrices were computed using Pearson's correlation coefficient at each of the three time points for individual patients (Fig. 2D). The concordance map was computed using the concordance correlation coefficient (concordance) (Lin, 2016) at the voxel-level across the three time points (Lohmann et al., 2012). Concordance values range between −1 and 1, such that lower concordance reflects larger alterations in the functional connectivity pattern over time.

### 2.8 The relationship between lesion location along connectivity gradients and changes in functional connectivity after stroke

Concordance values were correlated with distance-to-lesion values using Spearman's rank-order correlation coefficient (Fig. 2E). This analysis was repeated for each connectivity gradient separately. Positive correlations suggest that changes in functional connectivity are more pronounced in voxels that share similar connectivity patterns with the lesion.

### 2.9 The relationship between changes in functional connectivity over time and anatomical lesion location

Euclidean distances from each voxel to the infarct area in MNI152 (3 mm^3^) space using three-dimensional voxel coordinates were computed for each patient. The resulting anatomical distance values were correlated with concordance values (using Pearson's correlation coefficient). A regression analysis was applied to remove the contribution of this factor from concordance values. Residuals were correlated with gradient-based distance-to-lesion values (using Spearman's rank-order correlation coefficient).

### 2.10 The relationship between changes in functional connectivity over time and lesion size

Individual lesion size was computed for each patient based on the lesion masks obtained at the day of admission (see Section 2.5). The number of voxels in each mask was multiplied by the volume of a single voxel to obtain lesion volume. The resulting values were correlated with Spearman's correlation values obtained along each of the three gradients using Pearson's correlation coefficient.

### 2.11 The relationship between changes in functional connectivity along connectivity gradients and clinical scores

Two analyses were performed using the clinical scores data; an analysis testing for the link between changes in functional connectivity and changes in clinical scores (analysis 1), and an analysis to test whether individual differences between distance-to-lesion and changes in functional connectivity over time along individual gradients are associated with clinical status at admission and discharge separately (analysis 2). Given the exploratory nature of these analyses, the results are interpreted descriptively. *P*-values are reported, but we refrain from making statements about statistical significance because of the known issues with interpreting *p*-values in this way in exploratory analyses (Gelman and Loken, 2014).

Analysis 1: Individual gradients were divided into uniform parcels (bins). We varied the number of bins used for the parcellation from 5 to 3000 in order to classify parts of the gradients as affected by the lesion. At each bin number and for each stroke patient, bins that overlapped with lesioned-voxels were identified as “lesion-affected”, whereas the remaining bins were defined as “lesion-unaffected”. An overall delta-concordance measure was computed as the difference between average concordances in lesion-unaffected and lesion-affected bins, such that *delta* − *concordance* = μ_*unaffected*_ − μ_*affected*_. A positive delta-concordance value reflects a higher change in functional connectivity over time in affected bins. Of note is that lesioned voxels were removed from this computation, thereby the difference in concordance reflects the degree of preferential change in functional connectivity in affected yet structurally intact areas.

To explore the link between changes in clinical scores and the overall delta-concordance measure detected along gradients, the National Institute of Health Stroke Scale (NIHSS) was used. The NIHSS values were assessed at the day of admission (day 0) and discharge (day 5). Twenty-seven patients out of 28 completed the NIHSS assessment at both time points. Patients were divided into two groups; those whose clinical scores changed from day 0 to day 5 (“clinical change”, *n* = 16), and those who did not change (“no clinical change”, *n* = 11).

Permutation testing (with 10,000 iterations) was used to examine the significance of the difference in mean delta-concordance values for the two groups of patients (“clinical change” versus “no clinical change”). The test was repeated for each variation of bin numbers as well as for each of the three connectivity gradients. Positive values reflect that a preferential change in concordance over affected bins is more pronounced in patients who changed their clinical score from day 0 to day 5.

Analysis 2: To test if individual differences in the relationship between distance-to-lesion and changes in functional connectivity over time along individual gradients are associated with clinical status, we used a linear regression model. The slope and the intercept were obtained for the above relationship for each gradient in each patient. The slope reflects the degree of dependence between changes in functional connectivity over time and similarity with the lesion in terms of connectivity patterns - positive slopes indicate that changes in functional connectivity over time are higher (i.e. concordance lower) in areas that share similar connectivity patterns with the lesion (i.e. smaller distance to lesioned voxels in connectivity space). The intercept reflects the level of changes in functional connectivity over time in the areas functionally most similar to the lesion, with lower intercepts representing greater degrees of alteration.

The slope and the intercept values were then used to test for correlation with clinical status at admission and at discharge separately, using Kendall's Tau correlation coefficient. Clinical status was determined using two clinical scores: NIHSS and modified Ranking Scale (mRS). Although these two measures are correlated (Saver and Altman, 2012) the NIHSS measures the overall severity of the neurological deficit, while the mRS is a disability scale that measures functional impairment.

## 3 Results

### 3.1 Mapping stroke lesions onto connectivity gradients

**Fig. 3:**
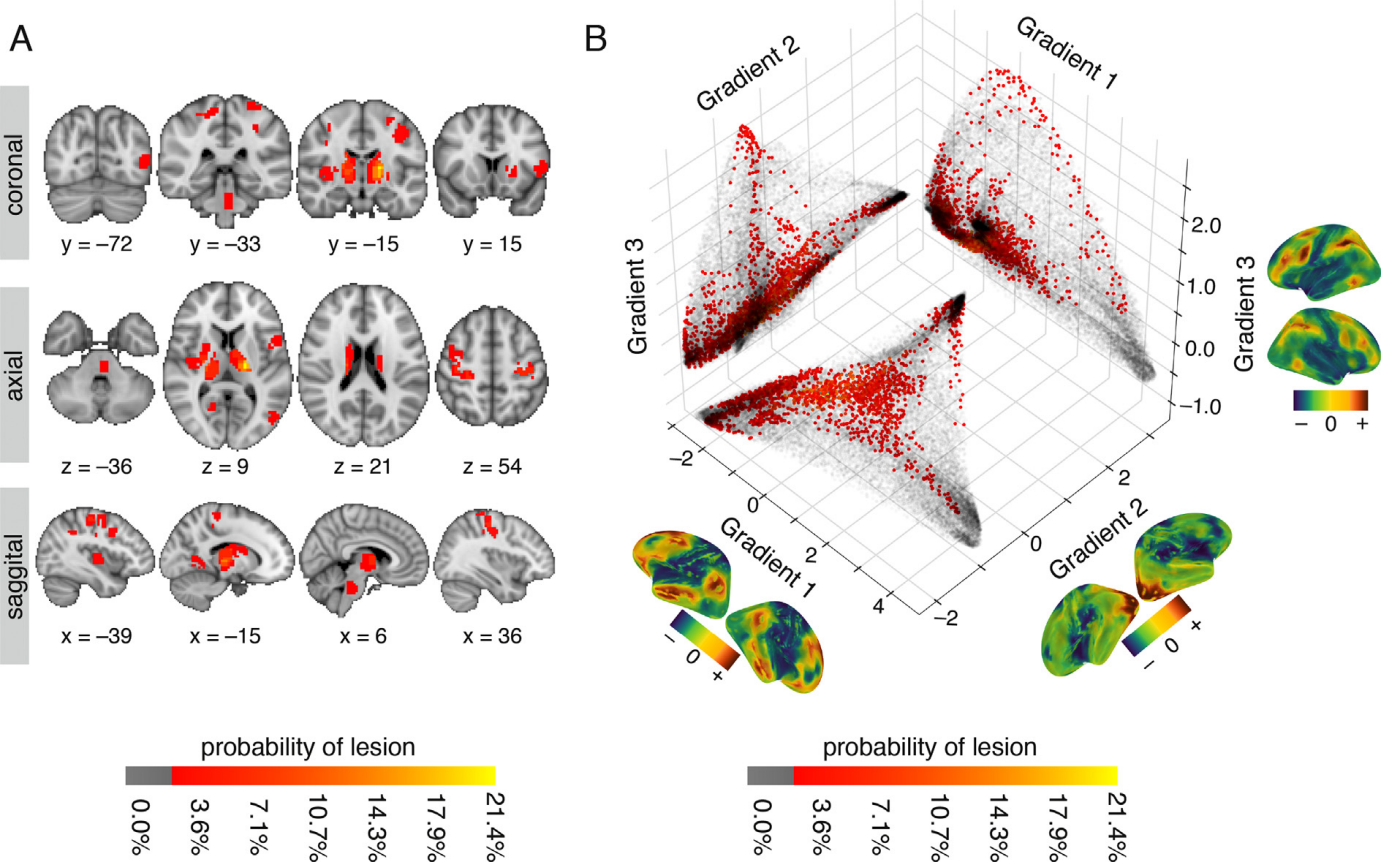
Lesion location across patients shown in anatomical space and along connectivity gradients (A) Anatomical lesion distribution in individual stroke patients (*n* = 28) projected onto an MNI brain. The red-to-yellow color bar indicates the percentage of patients with lesions in that voxel. (B) Location of lesions projected onto the first three connectivity gradients. The three connectivity gradients represent a low-dimensional description of the whole-brain connectivity matrix obtained using healthy controls' data (n = 28). Corresponding spatial maps of each connectivity gradient are projected on brain surface mesh near respective axes. Colors represent positive (sienna) and negative (dark blue) embedding values, in accordance with values along the axes. Along each gradient, voxels that share similar connectivity patterns are situated close to one another and have similar embedding values. Grey scatter plots depict a two-dimensional connectivity space created as a combination of any two given gradients. Lesion location along each gradient is projected onto the two-dimensional space as an alternative approach to anatomical lesion mapping. The red-to-yellow color bars indicates the percentage of patients with lesions in that voxel. Lesioned voxels are mostly clustered around the edges of the connectivity gradients such that they affect sensorimotor areas and ventral and dorsal areas associated with attention.

To map heterogeneous lesions across our sample of patients, individualized lesion masks were delineated and projected onto a standard MNI brain (Fig. 3A), as well as onto the first three connectivity gradients (Fig. 3B). Lesions were heterogeneous in both location and size (mean volume = 4.11 cm^3^, SD = 2.80 cm^3^), and distributed in subcortical (*n* = 13), cortical (*n* = 14), and brainstem (n = 1) regions. For further details on individual lesion location and affected vascular territories, see Supplementary Table 1.

Projecting lesion locations onto the connectivity gradients enabled us to assess which portions of connectivity space were affected by the stroke. The template connectivity space was based on a decomposition of voxelwise functional connectivity data from healthy controls. Voxels that share functional connectivity patterns are situated closer to one another along a given connectivity gradient. For example, voxels that are part of the default-mode network are clustered at the high end of Gradient 1, and those that are part of primary sensory areas at the low end (Margulies et al., 2016). Here, we used the first three gradients that account for a total variance of 50.84% in the healthy control connectivity data (see Supplementary Fig. S2).

Fig. 3B demonstrates the distribution of lesioned voxels within the three-dimensional connectivity space. We found that although the anatomical location of lesions was heterogeneous (Fig. 3A), within the connectivity space lesions were predominantly clustered at the extremes of each gradient, especially those of Gradients 1 and 3 (Fig. 3B).

### 3.2 The impact of lesion location along specific connectivity gradients on reorganization

To determine if the location of lesions along specific gradients is associated with changes in functional connectivity after stroke, we computed for each voxel: 1) concordance, which reflected the degree of change in the functional connectivity pattern over time. Concordance values range between −1 and 1 such that lower values reflect a larger change in functional connectivity pattern over time; and, 2) distance-to-lesion along each connectivity gradient., which reflects the similarity of a given voxel with the lesion in terms of connectivity patterns. Low distance values reflect voxels that share similar functional connectivity pattern with the lesion site. Importantly, the lesioned voxels were excluded from both these analyses such that only the indirect effects of the lesion (i.e., diaschisis) were assessed. Concordance and distance-to-lesion were correlated for individual patients, and individual connectivity gradients.

**Fig. 4:**
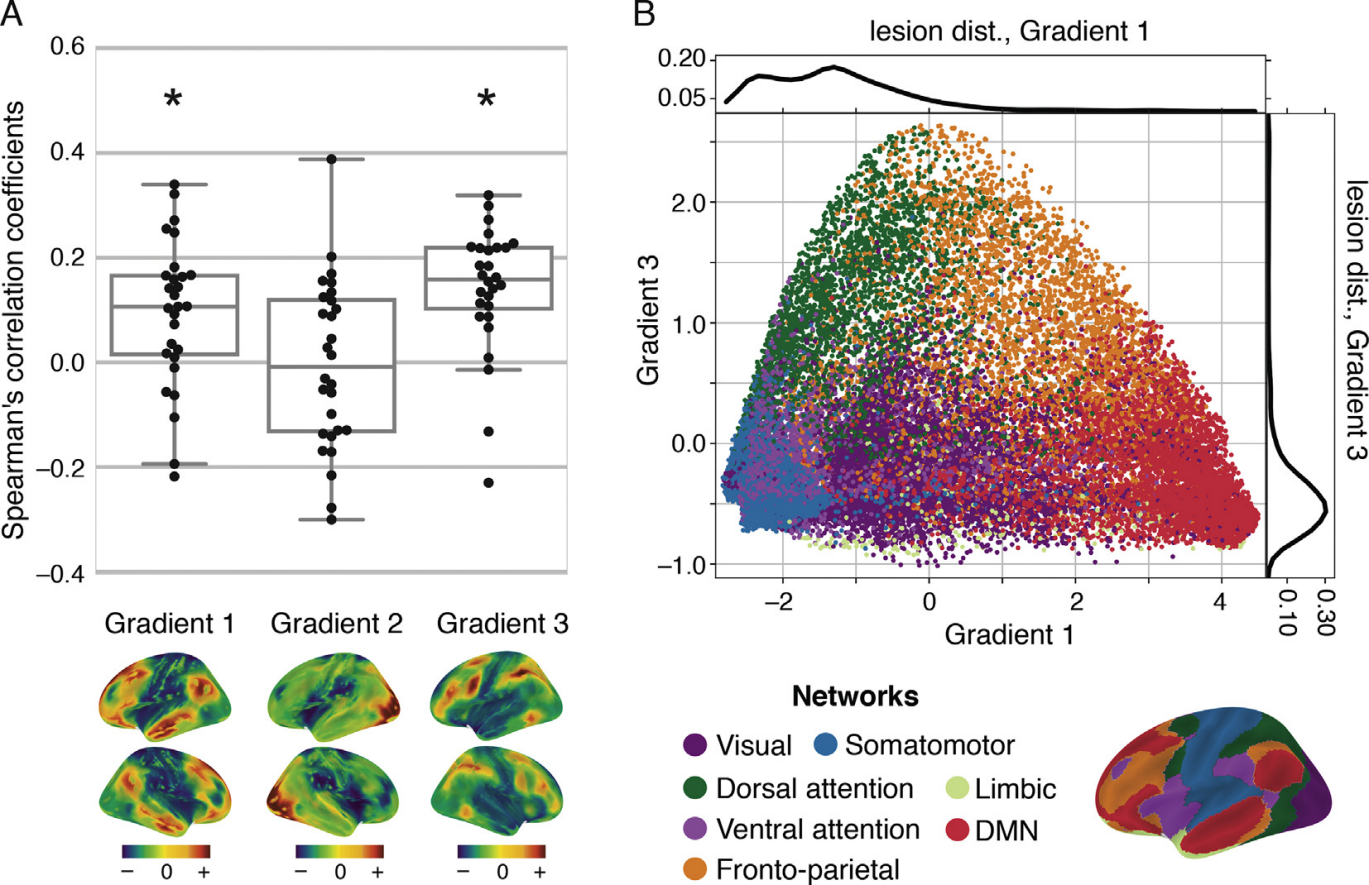
The relationship between voxelwise similarity to the connectivity patterns of lesioned areas and the degree of changes in functional connectivity in non-lesioned voxels over time. (A) Correlation values between distance-to-lesion and concordance (y-axis) are shown for individual patients and the three connectivity gradients (x-axis). The spatial map of each connectivity gradient is shown below the respective location on the x-axis. Correlations were significantly positive for Gradient 1 (*P* = .0027, W = 71.0, one-tailed Wilcoxon signed-rank test) and Gradient 3 (*P* = .0001, W = 35.0), but not for Gradient 2 (*P* = .76, W = 189.0). The more similar a voxel's connectivity pattern is to that of lesioned voxels on connectivity Gradients 1 and 3, the more pronounced its functional connectivity changes over time. (B) Continuous connectivity gradients and corresponding seven canonical resting-state networks (Thomas Yeo et al., 2011). Voxels are situated based on their embedding values along Gradient 1 (x-axis) and 3 (y-axis) and colored according to their network assignment. Gradient 1 captures the dissociation between the default-mode network (DMN) and the sensorimotor networks on its two edges, while Gradient 3 captures the dissociation between dorsal attention/fronto-parietal networks and sensorimotor/DMN networks on its two edges. Lesion distributions along connectivity gradients are overlaid on the individual gradient axes. Lesions overlap most frequently with the lowest ends of Gradients 1 and 3.

We found a significant relationship between the degree of change in functional connectivity over time and similarity between non-lesioned and lesioned voxels in terms of connectivity patterns along Gradient 1 and Gradient 3. No significant relationship was found for Gradient 2 (Fig. 4A, Table 1).

**Table 1:** Summary of statistical results.

	Gradient 1	Gradient 2	Gradient 3
r-values	[−0.22, 0.34]	[−0.30, 0.39]	[−0.23, 0.32]
median	0.11	−0.01	0.16
W	71.00	189.00	35.00
p-values	.0027*	.76	.0001*

W; Wilcoxon signed-rank test. * Statistically significant (p<0.05)

Fig. 4B demonstrates the correspondence between the connectivity space described by Gradients 1 and 3, and a canonical set of seven resting-state networks (Thomas Yeo et al., 2011). Gradient 1 captures the dissociation between the default-mode network (DMN) and the sensorimotor/visual networks, while Gradient 3 captures the dissociation between dorsal attention/fronto-parietal networks and sensorimotor/visual/DMN networks. For a descriptive analysis of the relationship between connectivity gradients and cognitive functions see Supplementary Material M4 and Supplementary Fig. S3.

Given the expected partial correlation between distance-to-lesion in connectivity space and anatomical distance, we further assessed whether anatomical location contributed to the relationship with connectivity space. We found a significant relationship between distance from the lesion in anatomical space and changes in functional connectivity over time (*P* = .0042, one-tailed Wilcoxon signed-rank test). However, using anatomical distance as a regressor of no interest did not alter the significance of our main result (see Supplementary Fig. S4). Functional connectivity therefore preferentially changes after stroke in voxels that share similar connectivity patterns to those of lesioned voxels along Gradients 1 and 3. This relationship cannot be solely explained by the anatomical distance from the lesion. Furthermore, the potential impact of lesion size on the relationship between distance-to-lesion and changes in functional connectivity over time was examined using a correlation analysis. We found no significant relationship between these two measures (see Supplementary Fig. S5).

### 3.3 Clinical relevance of functional connectivity alterations detected along connectivity gradients

Previous studies have linked alterations in functional connectivity with clinical trajectory (He et al., 2007; Ovadia-Caro et al., 2013; Park et al., 2011; Ramsey et al., 2016; van Meer et al., 2010), thereby supporting the functional significance of connectivity changes after stroke. We thus explored the relationship between changes in functional connectivity and patients' clinical trajectory for each connectivity gradient.

We tested for a group difference in concordance in affected yet structurally intact areas between patients who demonstrated a change in clinical scores from day 0 to day 5 and those who did not. A positive difference in the mean of the two groups reflects an association between preferential changes in functional connectivity in affected areas and a change in clinical scores over the first week after stroke. To assess the impact of binning on our results, we varied the number of bins used to divide the gradients into parcels of equal size (bin numbers ranged from 5 to 3000). We found no substantial difference between patients who changed in clinical scores and those who did not for any of the connectivity gradients, across different bin numbers. The averaged difference in mean for the two groups was 0.0014 (range: −0.004 to 0.015) for Gradient 1, 0.0095 (range: 0.003 to 0.015) for Gradient 2, and 0.011 (range: 0.0012–0.019) for Gradient 3. The range of corresponding *p*-values was .15 to .61 for Gradient 1, .12 to .4 for Gradient 2, and .03 to .46 for Gradient 3 (see Supplementary Fig. S6).

In an additional analysis, we examined if individual differences in the relationship between changes in functional connectivity over time along gradients are associated with the clinical status. To achieve this, we tested whether slopes and intercept values from the linear regression between distance-to-lesion and concordance correlate with clinical status at admission and discharge. We found a positive correlation between the slope and clinical status (using both scores) at discharge for Gradients 1 and 3, but not for Gradient 2. This positive relationship indicates that the bigger the difference between functional connectivity alterations in areas maximally similar and dissimilar (in terms of connectivity pattern) to the lesion in connectivity space, the worse the clinical status at discharge. A relatively high negative correlation was found between slope and NIHSS at discharge in Gradient 2, but this was much lower when the mRS was used. In addition, we found a negative correlation between the intercept and clinical status (using both scores) at discharge for Gradients 1 and 3, but not for Gradient 2. This negative relationship indicates that the lower the functional connectivity alterations in areas with connectivity patterns similar to the lesion, the better the clinical status at discharge. The linear correlation between slope/intercept and clinical score was stronger for clinical status at discharge than for clinical status at admission for all the performed tests (see Table 2, all results available here: https://doi.org/10.6084/m9.figshare.7680485).

**Table 2:** Kendall's Tau correlation coefficient values for the relationship between reorganization along connectivity gradients and clinical status.

Gradient		Admission		Discharge	
		mRS	NIHSS	mRS	NIHSS
1	Slope	0.08	0.06	0.33	0.44
	p-value	.60	.67	.04	.004
	Intercept	−0.09	−0.08	−0.24	−0.29
	p-value	.57	.58	.12	.05
2	Slope	−0.08	−0.14	−0.07	−0.23
	p-value	.60	.31	.68	.14
	Intercept	−0.03	−0.06	−0.13	−0.10
	p-value	.84	.67	.40	.49
3	Slope	0.05	0.09	0.28	0.30
	p-value	.73	.55	.07	.05
	Intercept	0.08	0.05	−0.23	−0.21
	p-value	.60	.73	.15	.17

## 4 Discussion

In this study, we found that stroke induces a gradual change in functional connectivity that follows specific connectivity gradients. Beginning with data acquired on the day of symptom onset, we showed that the degree of reorganization over the first week is influenced by the lesion location along connectivity Gradients 1 and 3. Voxels that have connectivity patterns similar to those of the lesion demonstrate a preferential change in functional connectivity over time (as measured by concordance), regardless of their anatomical distance from the lesion. We have implemented a decomposition approach that overcomes the necessity to parcellate the brain into discrete networks, retains information from single voxels and provides a data-driven template for studying reorganization at the connectome-level. This study therefore supports the biological rationale for gradients by showing that functional reorganization following stroke can be captured along specific dimensions of this continuous connectivity space.

Our results are in line with previous stroke studies that have used a priori defined networks. Diaschisis preferentially affects functional connectivity in affected networks, as has been shown for the sensorimotor, language and attention networks (Baldassarre et al., 2014; Carter et al., 2010; He et al., 2007; Ovadia-Caro et al., 2013; Siegel et al., 2016a, Siegel et al., 2016b; Wang et al., 2010; Warren et al., 2009). Here, we extend these findings to the continuous representation of the connectome. We demonstrate that reorganization, as reflected in functional connectivity alterations, changes as a function of the distance from the lesion along specific connectivity gradients. However, it is not exclusively restricted to the affected network. Thus, while the most pronounced changes take place in areas with connectivity patterns similar to the lesion, the effects of stroke gradually spread along specific dimensions of the connectome.

We found that connectivity Gradients 1 and 3 better predicted the impact of a lesion on functional connectivity than Gradient 2. The three connectivity gradients capture distinct connectivity axes, with different functional domains on their extremes. One crucial difference between these gradients is that Gradient 2, in contrast to the others, represents a spectrum of relatively local patterns of connectivity, spanning sensory and motor systems (see Supplementary Fig. S7). In Gradient 2, changes in functional connectivity over time were not related to the similarity between connectivity patterns in non-lesioned voxels and lesioned voxels. Interestingly, previous studies have shown that the value of functional connectivity for predicting deficits is domain-specific and that for motor and visual deficits, lesion topography is of higher predictive value than functional connectivity (Siegel et al., 2016a, Siegel et al., 2016b). Widespread effects of diaschisis could therefore be more pronounced in domains that involve multiple, higher-order regions to begin with, like attention and memory. Such a distinction between unimodal and heteromodal/multimodal regions is represented in Gradients 1 and 3 and could potentially explain why reorganization is captured only along these two dimensions. In general, it remains to be determined which types of post-stroke deficits are likely to benefit from the application of such a continuous approach to functional connectivity.

Our study demonstrates the importance of lesion location within connectivity space for understanding the reorganization of functional connectivity. However, the similarity in terms of connectivity patterns between non-lesioned and lesioned voxels is partially captured by anatomical distance, as areas close to one another are highly interconnected (Ercsey-Ravasz et al., 2013). In addition, local physiological changes in areas directly surrounding the lesion (Dirnagl et al., 1999) can also contribute to changes in functional connectivity (Khalil et al., 2017; Siegel et al., 2016a, Siegel et al., 2016b). We therefore calculated in a control analysis the Euclidian distances from each voxel to the infarct area using a three-dimensional anatomical space. We found a significant relationship between distance based on anatomy and changes in functional connectivity as measured by concordance. However, when regressing the contribution of this factor from our main analysis, the results remained consistent (see Supplementary Fig. S4). Consequently, changes in functional connectivity detected along connectivity Gradients 1 and 3 could not be solely explained by lesion topography or physiological processes occurring in the vicinity of the lesion site. This analysis emphasizes the significant contribution of functional connectivity changes in distant yet connected areas to the global process of reorganization.

Although the individual lesion area was removed from the main analysis, the potential impact of lesion size on changes in functional connectivity was additionally explored using a correlation analysis. No significant relationship was found for any of the three gradients between lesion volume and the magnitude of correlation between distance-to-lesion and changes in connectivity over time (See Supplementary Fig. S5). However, this lack of relationship can be explained by the homogeneity in lesion size in our specific cohort (4.11 ± 2.75 cm^3^, mean ± standard deviation). This analysis therefore does not exclude the possibility that such a relationship can be found in samples with a wider range of lesion sizes.

The conceptual shift from mapping localized brain regions to networks has provided a substantial improvement to how we understand the organization of functional and cognitive systems. Connectivity gradients are a further conceptual advance as they represent connectivity beyond segregated networks, thereby providing a macro-scale fingerprint of whole-brain connections (Mars et al., 2018). This approach is potentially useful for pathological states associated with widespread connectivity changes, or those in which localization of the pathology is ill-defined. Promising results have recently been reported in a large cohort of individuals with autism spectrum disorder (ASD), where alterations in the global hierarchy were captured along the first dimension of connectivity space (Gradient 1) (Hong et al., 2019). Specifically, individuals with ASD demonstrate divergent transition between sensory and higher order default-mode regions along Gradient 1, as compared with controls. Alterations in the hierarchical connectivity pattern captured along this dimension was associated with deficits in social cognition as well as low-level behavioral symptoms, thereby providing a unified framework for mitigating the diverse behavioral effects associated with this pathology (Hong et al., 2019).

Here we used stroke as a perturbation model to support the validity of connectivity gradients. While more studies are necessary to understand better the utility of this framework for stroke prognosis, the current findings provide support for conceptualizing brain connectivity within a continuous connectivity-defined space. In stroke, assessing reorganization using this approach has an important potential advantage; compared to discrete connectivity approaches, gradients preserve the role of connector hubs, which connect between sub-networks. These hubs are essential for intact modular topology (Gratton et al., 2012), have been linked to cognitive performance (Bertolero et al., 2018), and play an important role in post-stroke recovery (Siegel et al., 2017).

Ultimately, measures of network disruption or reorganization after stroke are expected to be associated with clinical deficits or clinical recovery. This has been shown for various measures of functional connectivity in previous studies (He et al., 2007; Ovadia-Caro et al., 2013; Park et al., 2011; Ramsey et al., 2016; van Meer et al., 2010). In an exploratory analysis that was beyond the primary aim of this study, we found no difference between patients with clinical change and patients without clinical change over the first week post-stroke (defined by the NIHSS) in terms of the changes in connectivity over time in affected as compared to non-affected parts of the continuous connectivity space. Of interest nevertheless is that for Gradient 2 and Gradient 3, group differences in these changes were not randomly distributed and were positive in value (see Supplementary Fig. S6).

In another exploratory analysis, we found that the relationship between changes in a voxel's functional connectivity and its similarity to the lesion in terms of connectivity was associated with the clinical scores at discharge (but not at admission). This result was more evident for Gradients 1 and 3 than for Gradient 2 (see https://doi.org/10.6084/m9.figshare.7680485). These results provide us with a starting point for investigating the clinical significance of functional connectivity alterations in the connectivity gradients framework. However, due to the exploratory nature of these analyses, as well as the lack of detail offered by the NIHSS and mRS in terms of assessment of functional deficits, these results should be verified in independent future studies. By using detailed clinical outcome measures as benchmarks, future studies could directly compare the value of discrete and continuous functional connectivity approaches. Given previous findings (Gratton et al., 2012; Siegel et al., 2017; Siegel et al., 2016a, Siegel et al., 2016b), it can be hypothesized that continuous approaches may be more predictive of outcome in patients with either functional deficits of a distributed nature (e.g., attention deficits) or those with lesions in hub areas.

Connectivity gradients can provide a framework to develop detailed models of whole-brain reorganization after stroke, either spontaneously or in response to interventions such as non-invasive brain stimulation. Currently, our models of reorganization after stroke are in many aspects network-limited, and the optimal way to deal with lesion heterogeneity across patients, or with deficits in multiple domains in an individual patient, remains unclear. In addition, current protocols for non-invasive brain imaging techniques are based on single network models (Di Pino et al., 2014; Morishita and Hummel, 2017; Ovadia-Caro et al., 2019) although widespread effects of stimulation have been reported in healthy controls (Antonenko et al., 2017; Dijkhuizen et al., 2014; Sehm et al., 2012). Connectivity gradients could be potentially used to refine our models of reorganization in response to a lesion, extending our description of diaschisis effects to the connectome level.

## 5 Conclusions

Connectivity gradients offer a methodological advancement in the representation of whole-brain connectivity patterns. By demonstrating that widespread effects of localized lesions follow the architecture captured along specific dimensions of this space, we provide empirical evidence supporting the biological validity of this approach, and suggest a novel framework to characterize stroke reorganization and recovery.

## Funding sources

This work was supported by the German Federal Ministry of Education and Research via the grant center for Stroke Research Berlin (01EO0801 and 01EO01301), the European Union Seventh Framework Program [FP7/2007–2013] under grant agreement no. 278276 (WAKE-UP) for J.B.F, and the Einstein Foundation Berlin (Grant No. EDP-2016-318) for S.O.C.

## Declaration of Competing Interest

J. B. F. has received consulting, lecture, and advisory board fees from BioClinica, Cerevast, Artemida, Brainomix, Merck and Lundbeck. J.B·F., A.K.K., and K.V. are the co-inventors of European Patent application 17179320.01-1906.

## Appendix A Supplementary data

Supplementary material 1 Image 1
